# An industry perspective on the use of “atoxigenic” strains of *Aspergillus flavus* as biological control agents and the significance of cyclopiazonic acid

**DOI:** 10.3109/15569543.2011.588818

**Published:** 2011-07-19

**Authors:** Eileen D King, Albeit B (Bobby) Bassi, David C Ross, Bernd Druebbisch

**Affiliations:** Syngenta Crop Protection, LLC, Greensboro, NC, USA

**Keywords:** Aflatoxin, mycotoxins, competitive exclusion, Afla-Guard®, AF36, K49

## Abstract

Several nonaflatoxigenic strains of *Aspergillus flavus* have been registered in the United States to reduce aflatoxin accumulation in maize and other crops, but there may be unintended negative consequences if these strains produce cyclopiazonic acid (CPA). AF36, a nonaflatoxigenic, CPA-producing strain has been shown to produce CPA in treated maize and peanuts. Alternative strains, including Afla-Guard® brand biocontrol agent and K49, do not produce CPA and can reduce both aflatoxin and CPA in treated crops. Chronic toxicity of CPA has not been studied, and recent animal studies show significant harmful effects from short-term exposure to CPA at low doses. Grower and industry confidence in this approach must be preserved through transparency.

## Introduction

Biological control is certainly not a new concept, having been practiced for many years for control of various weeds, insects, nematodes and diseases of economic importance in agriculture, and in turf, lawn, and garden settings. In most instances, the organism applied as a biological control agent belongs to a different taxonomic group from the target pest. Recently, however, attempts have been made to use “atoxigenic” strains of the fungal species *Aspergillus flavus* Link to control naturally occurring, aflatoxin-producing “toxigenic” strains. These efforts have met with significant success, and several nonaflatoxigenic strains of *A. flavus* have been patented, registered, and commercialized in the USA for aflatoxin reduction in crops such as cotton, peanuts, and maize (Dorner et al., 1992, 1999, 2000; Antilla and Cotty, 2002; Dorner, 2004a, 2004b). Afla-Guard® biocontrol agent, containing the nonaflatoxigenic *A. flavus* strain NRRL 21882, received US Environmental Protection Agency (US EPA) registration for use on peanuts in 2004 and on maize in 2008, becoming the first such product commercialized for use on this major food and feed crop anywhere in the world. AF36 (NRRL 18543), sponsored by the nonprofit Arizona Cotton Research and Protection Council and registered in 2007 for use on cotton in the western USA, has recently received US EPA registration for use on maize in Texas and Arizona. More recently, K49 (NRRL 30797) was patented by the USDA. These same nonaflatoxigenic strains, or similar strains, are under development in Brazil, Africa, China, and other parts of the world. These strains are believed to act by “competitive exclusion” of the native strains, thereby reducing the amount of aflatoxin in the harvested crop (Cotty, 1990; Dorner et al., 1992, 1999, 2000; Cotty and Bayman, 1993; Dorner, 2004a, 2004b). In order to do this, the field must be inoculated with conidia of the nonaflatoxigenic strain at the right stage of crop development and these conidia must go on to successfully establish a strong presence on the crop and in the ecosystem within the field. To date, this has been accomplished by applying the nonaflatoxigenic strain to the field along with a grain-based carrier, which also serves as a food source for the introduced fungal strain.

While nonaflatoxigenic strains of *A. flavus* may offer a simple and effective means for reduction of aflatoxin during field production, it must be recognized that all “atoxigenic” strains are not created equal and that, in some cases, the name may be misapplied. It is important to note that, in addition to the aflatoxins B1 and B2, many strains of *A. flavus* are capable of producing cyclopiazonic acid (CPA), an indole tetramic acid mycotoxin first isolated from *Penicillium cyclopium* (Holzapfel, 1968; Luk et al., 1977; Gallagher et al., 1978, Dorner et al., 1983; Horn and Dorner, 1999). Though CPA has been implicated as a probable co-contaminant with aflatoxin in peanut meal associated with the Turkey “X” disease outbreak of 1960 (Cole, 1986; Bradburn et al., 1994; Richard, 2008), this toxin has received much less attention and has been the subject of many fewer studies than aflatoxin. Despite a slowly accumulating body of knowledge about its toxicity to various animal species and its mechanisms of toxicity, CPA is currently not regulated by the US Food and Drug Agency (FDA) or similar government regulatory agencies in other countries. There are few available studies and no routine surveys for the presence of CPA in grains or other commodities, in part because of the lack of regulatory requirements for testing, the relative difficulty of the analytical methods used in its detection (Moldes-Anaya et al., 2009; Diaz et al., 2010), and probably also in part because many researchers have concluded, despite the dearth of data, that CPA is a relatively “benign” toxin, that it is not likely to be present in sufficient concentrations in food or feed to pose a significant problem or that efforts to reduce aflatoxin contamination will indirectly result in reductions in CPA contamination (Byrem et al., 1999; Burdock and Flamm, 2000; Chang et al., 2009a). Other researchers have expressed concern that the potential for harm to humans or animals from exposure to CPA has not been adequately evaluated or addressed (Stoltz et al., 1988; Dorner et al., 1994; Kubena et al., 1994; Balachandran and Parthasarathy, 1996b; Prasongsidh et al., 1997; Kumar and Balachandran, 2009) and several studies have demonstrated the presence of CPA in food and feed items sampled from various locations around the world, sometimes at levels in the range of 2.8 to 12 μg/g (Stolz et al., 1988; Widiastuti et al., 1988; Urano et al., 1992; Balachandran and Parthasarathy, 1996b). This risk becomes more serious when CPA-producing “atoxigenic” strains are intentionally applied to a major food and feed crop such as maize, under conditions that favor colonization of the plant by the introduced strain.

Dorner et al. (2000) clearly demonstrated the accumulation of CPA in peanuts treated with AF36. The recent publication of a study comparing the accumulation of aflatoxin and CPA in maize treated with several different toxigenic and “atoxigenic” strains under field conditions (Abbas et al., 2011) provides clear confirmation that AF36 can produce CPA in maize. In this review, we discuss the differences among several “atoxigenic” strains currently in development or commercial use, the available information regarding CPA toxicity in humans, dogs, swine, cattle, and chickens, the limited information regarding CPA in food and feed items, and the available information regarding CPA transfer and persistence in meat, milk, and eggs. Finally, we present an industry perspective on standards for selection of an “atoxigenic” strain and the need for full transparency for growers and downstream stakeholders regarding these strains.

## Genetic basis of “atoxigenicity”—differences among strains

Enzymes and regulatory proteins for aflatoxin synthesis in *A. flavus* and *A. parasiticus* are encoded by more than two dozen clustered genes in a 66kb region on chromosome 3 (Yu et al., 2004; Ehrlich et al., 2005). In a study of genetic variation among 38 nonaflatoxigenic strains of *A. flavus*, Chang et al. (2005) demonstrated that the genetic basis for a specific strain's inability to produce aflatoxin might vary from a single-point mutation or small deletion to deletions larger than 45kb and involving multiple genes, concluding that these genetic defects could be grouped into eight typical deletion patterns. In pattern H, the most extensive deletion pattern observed, genetic material on both sides of the aflatoxin gene cluster was also deleted, and it was shown that the “right” side deletion extends to the *hexA* gene in the downstream sugar utilization gene cluster. Chang et al. (2009b) later confirmed that some of the genes required for production of CPA are located close to the aflatoxin gene cluster in *A. flavus* and that the strains with the largest deletion pattern have lost these genes for CPA production along with the aflatoxin gene cluster. NRRL 21882 (the strain contained in Afla-Guard® biocontrol agent) is one of the strains showing this very large deletion pattern, completely removing its ability to produce either aflatoxins or CPA. In addition, the extensive deletions in the aflatoxin and CPA gene clusters identified in NRRL 21882 are expected to serve as a safeguard against adverse genetic reversion or recombination which might restore toxigenicity (Chang et al., 2005).

In contrast, AF36 has only a single nucleotide change in the polyketide synthase gene (pksA) required for aflatoxin synthesis, which is sufficient to interrupt the coding sequence for this enzyme (Erlich and Cotty, 2004). This effectively removes the strain's ability to produce aflatoxins but has no impact on its ability to produce CPA. As previously noted, it has been confirmed that AF36 is able to produce CPA but not aflatoxins (Dorner et al., 2000; Abbas et al., 2011). Fortunately, this defect in the *pksA* gene affects the early part of the aflatoxin synthetic pathway, so accumulation of potentially toxic precursors to aflatoxin should not be an issue with this strain.

This type of genetic analysis has not been published for other strains known to be under testing or development for commercial use in the USA.

## CPA toxicity in animals

Studies to evaluate the mammalian and avian toxicity of CPA have been conducted in various species including rats (Purchase, 1971; Morrissey et al., 1985; Norred et al., 1985), mice (Nishie et al., 1987), chickens (Dorner et al., 1983; Norred et al., 1988; Kubena et al., 1994; Balachandran and Parthasarathy, 1996a; Gentles et al., 1999; Kamalavenkatesh et al., 2005, Venkatesh et al., 2005; Kumar and Balachandran, 2009; Malekinejad et al., 2010), dogs (Nuehring et al., 1985), and pigs (Lomax et al., 1984), and several comprehensive reviews of the toxicology are available (Burdock and Flamm, 2000; Chang et al., 2009a). At this time, there are no published studies regarding chronic toxicity of CPA in any animal species, and there are very few toxicity studies of any kind in most species.

In brief, CPA acts to specifically inhibit sarcoplasmic or endoplasmic reticulum calcium-dependent ATPase (SERCA), thus altering intracellular calcium flux. This disrupts the muscle contraction-relaxation cycle, resulting in increased muscle contraction. SERCA also is responsible for maintenance of the proper calcium gradient in cells, which is critical for cell proliferation, differentiation, and cell death. Several researchers have also suggested that CPA may be directly toxic to lymphocytes and lymphoid organs, such as thymus and spleen (Nuehring et al., 1985; Kamalavenkatesh et al., 2005; Venkatesh et al., 2005; Kumar and Balachandran, 2009), and that CPA, even at low doses, may induce inflammation in liver and kidney through oxidative stress (Malekinejad et al., 2010).

Dogs are sensitive to CPA, with 100% mortality occurring before the end of a 90-day study in groups dosed with 1.0 or 2.0 mg CPA/kg body weight/day (Nuehring et al., 1985). The dogs given the highest dose began to show clinical signs of intoxication within 2 to 4 days after the start of dosing and had either died or been humanely killed within 48 h of the onset of these clinical signs. These dogs rapidly progressed from anorexia to vomiting, diarrhea, pyrexia, dehydration, and CNS depression. In the highest dose group, with as few as 3 or 4 to 6 days' dosing before death, the researchers not only found extensive damage to the alimentary tract and kidneys but also found gross lesions in adrenal glands, skin, epididymis, uterus, and urinary bladder in some dogs. Microscopic analysis confirmed dose-related vascular damage, ulceration, necrosis, and nuclear enlargement in multiple systems. Largely similar gross pathology and histopathology were observed in the dogs dosed at 1.0 mg/kg/day, whereas fewer gross and microscopic lesions were observed in dogs dosed at 0.5 mg/kg/day. Whereas vascular damage was thought to be the primary event in the development of most of the necrotic lesions observed, damage to lymphoid organs and reduction in lymphocytes was thought to occur as a direct effect of CPA, perhaps through inhibition of DNA or protein synthesis. A NOEL of 0.1 mg/kg body weight/day and a LOEL of 0.5 mg/kg body weight/day can be derived from this 90-day study.

Pigs are also quite sensitive, with a NOEL between 0.01 and 0.1 mg/kg body weight/day in a 14-day feeding study in weaned pigs, and a LOEL of 0.1 mg/kg body weight/day (Lomax et al., 1984). Clinical signs were observed in pigs dosed at 1 or 10 mg/kg body weight/day, and these were more rapid in onset and more severe in pigs that had been fed the higher dose. Pigs dosed at 10 mg/kg body weight/day had roughened hair coats and displayed weakness, inactivity, and inappetence by the end of the 1st week, and this treatment group actually lost average body weight over the 14-day study period in each of the two study replicates (losses of 16% and 22.5% of initial average body weight in replicate 1 and 2, respectively). Most of these pigs passed yellow-brown to blood-tinged fluid feces in the 2nd week of the study. In each replication of this study, there was a 25% mortality rate in the 10 mg/kg body weight/day dose group by day 13 of dosing. As in dogs, CPA caused extensive damage to the alimentary tract of pigs given the highest dose, and gross lesions of the liver and kidneys were also observed in some of the animals in this dose group. The gross lesions observed at the highest dose were confirmed by microscopic observation, which showed mucosal necrosis in the stomach, diffuse severe lesions in the small and large intestines, and hepatic and renal lesions of varying severity among the individual animals. Clinical signs in the group dosed at 1 mg/kg body weight/day consisted of roughened hair coats and inactivity, and the number and severity of gross and microscopic lesions was much lower in this group, and still lower in the 0.1 mg/kg/day dosing group, in which clinical signs were not observed.

There is little information about the toxicity of CPA to other large farm animals, but incidents of “kodua poisoning” have been reported in India among cattle which ingested contaminated feed of *Paspalum scrobiculatum* (Bhide, 1962; Nyak and Misra, 1962), with symptoms including nervousness, staggering gait, lack of coordination, spasms, and depression; normally clearing up within 1 to 3 days, but occasionally resulting in death. Rao and Husain (1985) later demonstrated that this kodua poisoning was likely caused by CPA. Dorner et al. (1994) administered CPA to lactating ewes at a rate of 5 mg/kg body weight/day for 2 consecutive days. Within 24 h of the initial dose, milk production and feed intake had decreased substantially and, within 48 h, milk production had fallen to 20% of normal. The ewes had increased respiration rates and body temperatures, and dosing was discontinued for humane reasons. The ewes recovered and milk production returned to near-normal levels within 7 to 10 days.

More studies of CPA toxicity have been performed in chickens and other avian species than in most other animal species, again demonstrating significant sensitivity to CPA. In an acute toxicity study (Norred et al., 1988), a single dose of CPA at 0.5, 5.0, or 10.0 mg/kg body weight administered by gavage to 4-week-old chickens resulted in significant reduction in body weight gain at the two lower doses and actual body weight loss in the 10-mg/kg dosing group, and these effects were seen within 24 h of dosing in each group. Recovery of normal body weight gain was dose dependent, with the 0.5-mg/kg group recovering within 48 h of dosing, and the 5.0-mg/kg group recovering within 96 h, but the 10 mg/kg group continuing to show significantly reduced body weights vs. controls at the final, 96 h, sampling time. This study suggests that the acute NOEL in young chickens is less than 0.5 mg/kg body weight/day.

A second acute study (Dorner et al., 1994), in which laying hens were orally dosed with CPA at 2.5, 5.0, or 10.0 mg/kg body weight/day for 9 consecutive days, also showed rapid onset and dose dependency of effects. All hens in the 10-mg/kg group and 80% of hens in the 5-mg/kg group died before the end of the study, and egg production ceased 1 and 4 days after the initiation of dosing in the 10-mg/kg and 5-mg/kg groups, respectively.

In a recent study using multiple dose levels, Malekinejad et al. (2010) found significant effects in liver and kidney of broiler chickens after 28 days' exposure to CPA at dosages of 0.01, 0.025, and 0.050 mg/kg body weight/day, though no significant reductions in body weight gain or other clinical symptoms were observed. Increased liver weights and liver/body weight ratios were observed in chickens dosed at 0.025 or 0.050 mg CPA/kg body weight/day. Pathological abnormalities indicative of inflammation were observed in liver and kidney at all dose levels tested. Changes in numerous biochemical markers in blood serum which are associated with oxidative stress were observed in the two higher dose levels, and many of these changes were already evident after only 2 weeks of dosing. This study suggests that the NOEL is less than 0.01 mg/kg body weight and establishes a LOEL of 0.01 mg/kg body weight/day for CPA in chickens, much lower than previous studies.

In many of the other published studies in chickens, CPA was added to feed at a single, fixed concentration (ranging from 10 to 50 ppm in feed) and chickens were allowed to consume this feed *ad libitum* for periods of 21 to 28 days (Dorner et al., 1983; Kubena et al., 1994; Balachandran and Parthasarathy, 1996a; Gentles et al., 1999; Kamalavenkatesh et al., 2005, Venkatesh et al., 2005; Kumar and Balachandran, 2009). Effects observed in these studies included body weight reductions, where feed contained 25 ppm CPA or higher, and gross damage to liver, kidney, crop, and proventicular mucosa, with associated histopathological damage. Several more recent studies (Kamalavenkatesh et al., 2005; Venkatesh et al., 2005; Kumar and Balachandran, 2009) also documented damage to thymus and spleen, with increased apoptosis in splenocytes and reductions in lymphocytes, including helper and cytotoxic T cell populations, when chickens were fed *ad libitum* with feed containing CPA at 10 or 20 ppm. These findings suggest an immunosuppressive potential for CPA which, as Nuehring et al. (1985) suggested in dogs, may be the result of direct toxicity of CPA to lymphoid organs and endoplasmic reticulum (ER) stress.

In addition to the previously mentioned arguments supporting the hypothesis that CPA was involved in the Turkey “X” disease, there are other examples of clinical effects of CPA exposure in birds. An outbreak of disease in quail in Indonesia which was observed to have many of the characteristics of mycotoxicosis was investigated by Stolz et al. (1988), and a sample of the feed involved was found to contain CPA at 6000 ng/g, along with lower levels of aflatoxins (465 ng/g) and ochratoxin A (500 ng/g). The clinical signs in affected birds, including opisthotonus, as well as the histopathological findings support a diagnosis of CPA toxicity.

## CPA transfer to meat, milk, and eggs

Several studies have demonstrated that CPA is rapidly distributed into meat, eggs, and milk. CPA was shown to distribute rapidly into breast and thigh muscle of chickens after a single oral dose, with the peak concentration of CPA in the meat seen at 3 h after dosing (Norred et al., 1988). In the two lower doses used in this study (0.5 and 5.0 mg/kg body weight), CPA was eliminated from the meat within 24 to 48 h, whereas in birds receiving a single 10-mg/kg dose, the rate of elimination was slower, with the slowest elimination observed in birds with the most severe body weight reductions.

In a second short-term study (Dorner et al., 1994), laying hens were orally dosed with CPA at 2.5, 5.0, or 10.0 mg/kg body weight/day for 9 consecutive days. CPA began to appear in eggs from dosed hens within 24 h of the initial dose, accumulating almost exclusively in egg whites. In the group dosed at 2.5 mg/kg, the only dosing level in which egg production continued for the duration of the study, the CPA concentration in egg whites gradually increased over the first 6 days of the trial, with some variability thereafter. Concentration of CPA in pooled egg whites from this dosing group was 313 ng/g and 350 ng/g on day 6 and day 9, respectively.

A paired subchronic exposure study (Dorner et al., 1994), in which laying hens were dosed for 28 days at dosages of 1.25 and 2.5 mg CPA/kg body weight/day, again showed that most of the CPA in eggs accumulated in the whites, with variable concentrations over the course of the study, but with concentrations in the range of 60-160ng/g (mean =105 ng/g) in the 1.25 mg/kg/day dosing group and 18-193 ng/g (mean = 97 ng/g) in the 2.5 mg/kg/day dosing group.

In the third part of this study, Dorner et al. (1994) orally administered CPA to lactating ewes at a rate of 5 mg/kg body weight/day for 2 consecutive days. Within 24 h after the first dose, CPA concentration in milk averaged 236 ng/g, rising to a peak concentration of 568 ng/g on the day after administration of the second dose. The average CPA concentration declined to 262 ng/g by day 4 and was completely cleared from the milk by day 9, at which time the ewes had also fully recovered from the observed toxic effects.

Studies show that CPA remains quite stable in milk during normal storage and processing (Prasongsidh et al., 1997, 1998). CPA level in homogenized, pasteurized milk stored at 4°C was only reduced by 2.8%, 2.9%, and 5.8% after 7, 14, and 21 days, respectively. Freezing of homogenized, pasteurized milk yielded similar results, with reductions of 1%, 4.1%, and 5% after 7, 14, and 21 days, respectively, and, although the concentration continued to slowly decline thereafter, there was only a 10.8% reduction in CPA concentration after 140 days' storage. Freeze-drying produced similar results to freezing. More aggressive heat treatments (2 h at 100°C) resulted in some additional degradation of CPA, but 40% to 50% of the original concentration still remained intact. It was concluded that normal commercial processing methods would result in little removal of CPA from milk and milk products.

## CPA occurrence in food and feed items

There are relatively few reports of attempts to quantify CPA contamination of food and feed items. Gallagher et al. (1978) were the first to publish proof of the natural occurrence of CPA in maize, estimating the CPA concentration in one of the tested samples at 10 μg/g. Widiastuti et al. (1988) detected CPA in 21 of 26 corn samples collected from a poultry feed mill in Indonesia over the course of a year, with concentrations ranging from 0.03 to 9 μg/g, half with levels above 1 μg/g. In this study, the levels of CPA fairly consistently exceeded the levels of total aflatoxin, often by more than 100-fold, and all CPA-contaminated samples were co-contaminated with aflatoxin. Lee and Hagler (1991) analyzed maize stream-sampled from seven truckloads in North Carolina and found aflatoxin contamination in all seven loads, with concentrations ranging from 3 to 508 ng/g. They also found co-contamination with CPA in four samples, with concentrations ranging from <25 ng/g (the limit of determination) to 250 ng/g. Urano et al. (1992) analyzed 45 samples of maize collected before harvest from fields in Georgia and found that 51% of the samples had measurable levels of CPA (limit of determination 25 ng/g), with the highest concentration measured at 2.8 μg/g and an average concentration of 467 ng/g. All of these samples were co-contaminated with aflatoxin, while 16 samples (36%) contained only aflatoxin, and 6 samples (13%) were not measurably contaminated with either CPA or aflatoxin. Balachandran and Parthasarathy (1996b) examined multiple feed items collected in Tamil Nadu, India, including 20 randomly collected lots of maize and six lots known to be contaminated with aflatoxin. Nine of the 20 randomly sampled lots and one of the six aflatoxin-contaminated lots contained measurable amounts of CPA, with estimated levels ranging from 0.4 to 12 μg/g. Abbas et al. (2008) reported at-harvest CPA levels of 61 and 72.2 ng/g in Bt and non-Bt maize, respectively, a nonsignificant difference. All plots were co-contaminated with aflatoxin, and total aflatoxin levels were 104 and 200 ng/g in Bt and non-Bt maize, respectively. CPA has also been identified in samples of maize silage in Pennsylvania (Mansfield et al., 2008), though at relatively low concentrations. This was thought to be the result of colonization by *Penicillium spp.*

Oliveira et al. (2006) published the first report of CPA occurrence in milk in Brazil. CPA was detected in two samples (4.2% of the samples tested) of grade A milk at levels of 6.4 and 9.7 μg/L. CPA has been reported in other human foods and animal feeds, including peanuts, cheese, wheat, millet, rice, sunflower, sorghum, and tomato pulp and puree (Lansden and Davidson 1983; Urano et al., 1992; Balachandran and Parthasarathy, 1996b; Da Motta and Soares, 2001).

Measurement of CPA in these complex matrices has proven to be difficult, until recently requiring time-consuming clean-up procedures and intensive chemical usage but resulting in relatively high limits of determination and poor reproducibility (Moldes-Anaya et al., 2009). Recent studies have also demonstrated that CPA is unstable after heating in methanol-water solution, reacts with ambient oxygen, is adsorbed to plastic, and is unstable under certain acidic conditions (Diaz et al., 2010). Gallagher et al. (1978) also reported that metal chelate forms of tetramic acids like CPA may not react properly in spectroscopic analysis. This suggests that CPA concentrations in sampled material may have been systematically underestimated under earlier methodology.

## CPA in food and feed treated with “atoxigenic” strains

Abbas et al. (2011) studied the accumulation of aflatoxin and CPA in maize in field trials in which maize ears were “pin-bar” inoculated with one of three “atoxigenic” strains (AF36, K49, or NRRL 21882) alone or in 1:1 mixture with one of two aflatoxin and CPA-producing strains (K54 and F3W4). Control plots, inoculated with each of the two toxigenic strains alone were also included, as well as a noninoculated control. Samples analyzed 20 days after inoculation with K54 or F3W4 alone had CPA concentrations of 364 and 127 μg/g, respectively. Maize inoculated with AF36 alone was found to contain 190 μg/g of CPA, but CPA was not detected in maize inoculated with either NRRL 21882 or K49 alone or in the noninoculated control. When AF36 was coinoculated with K54 or F3W4, CPA levels were 139 and 143 μg/g, respectively, not statistically different from the level observed in maize inoculated with AF36 alone. Maize coinoculated with NRRL 21882 and K54 or F3W4 contained 21.5 and 20.8 μg/g CPA, respectively, whereas maize coinoculated with K49 and K54 or F3W4 contained 11.7 and 7.7 μg/g, respectively, with no statistically significant differences among these four treatments.

This study confirms that AF36 produces CPA in maize under field conditions and that it is not effective in reducing CPA accumulation when coinoculated with toxigenic strains. In contrast, K49 and NRRL-21882 produced no CPA when applied alone, and both were able to significantly reduce the amount of CPA contamination when coinoculated with either of the toxigenic strains tested. All three “atoxigenic” strains were effective in reducing aflatoxin contamination although K49 and NRRL 21882 were statistically superior to AF36 in this regard. While the “pin-bar” inoculation method and the analysis of only the inoculated kernels resulted in very high concentrations of the mycotoxins, the method allowed a controlled evaluation of relative competitiveness and toxin production or reduction by different strains.

Elevated levels of CPA were detected in peanuts treated with AF36 in a field trial comparing AF36 with NRRL 21882 (Dorner et al., 2000). CPA levels in edible peanuts treated with AF36 averaged 69.8 ng/g, which was significantly higher than the average of 1.7 ng/g observed in the untreated controls. CPA was not detected in peanuts from any of the plots treated with NRRL 21882. CPA levels in all peanuts averaged 321.9, 5.3, and 8.7 ng/g from plots treated with AF36, NRRL 21882, and in the untreated controls, respectively. As in maize, NRRL 21882 was capable of reducing both aflatoxin and CPA versus the controls, but AF36 reduced aflatoxin while increasing CPA by approximately 40-fold vs. levels in the untreated controls. AF36 is not currently registered for use on peanuts.

## Maize as a component of animal diets

Maize is an important component of food/feed for dogs, cattle, swine, chickens, and other livestock. Commercial dog food compositions are not available to the public, but many well-known brands of dog food list whole grain corn, ground whole corn, or corn meal as the No. 1 or No. 2 ingredient on their product labels. In some cases, corn gluten meal is also added as the No. 3 or No. 4 ingredient. Rations for finishing beef cattle are often composed of 60-96% shelled corn (Lalman and Sewell, 1993; Siemens et al., 1999) and rations for dairy cows may contain 65-70% shelled corn (Dunham and Call, 1989). Maize is a major component of feed for swine, increasing from 40% of the feed for 4-week old pigs to over 80% of the feed by the 20th week of life (Carr, 1998; Brendemuhl and Myer, 2009). Corn represents 55 to 71% of typical feed rations for broiler chickens, increasing with age of the chickens (Firman, 1993).

Using the LOEL of 0.01 mg/kg body weight/day derived from Malekinejad et al. (2010) for broiler chickens, it can be calculated that use of maize contaminated with CPA at levels between 0.14 and 0.23 μg/g in standard feed rations would be sufficient to reach this LOEL, and use of maize contaminated with 0.69 to 1.15 μg/g would expose the chickens to a daily dose of 0.05 mg/kg body weight/day, the highest dose tested in this study. This higher dose was associated with numerical but nonsignificant body weight gain reductions after 4 weeks, significant pathological changes to liver and kidney and significant changes in serum biochemical indicators indicative of oxidative stress. As the levels of CPA reported in the few available surveys summarized above suggest that it is not uncommon to see naturally occurring levels above 1 μg/g, and even up to 10-12 μg/g in maize, reduction of CPA contamination could have beneficial effects on chicken production.

Similarly, inclusion of maize contaminated with 3 to 6 μg/g CPA in standard rations, as described above, would be sufficient to exceed the NOEL for swine, and use of maize contaminated with 6 to 12 μg/g CPA would likely exceed the NOEL for many dogs.

## CPA toxicity in humans

There is very little evidence for human toxicity due to consumption of CPA-contaminated food, although Rao and Husain (1985) reported the isolation of CPA from two batches of kodo millet (*Paspalum scrobiculatum*) grain associated with incidents of “kodua poisoning” in humans and cattle in India. They further demonstrated that CPA was produced by strains of *A. flavus* and *A. tamarii* associated with these contaminated batches of millet. Unfortunately, the concentration of CPA in the affected millet was not determined.

Efforts have been made to estimate an acceptable daily intake (ADI) for humans, based on studies in test species. Burdock and Flamm (2000) proposed an ADI of 10 μg/kg body weight based on a NOEL in the range of 1.0 mg/kg/day from the pig study (Lomax et al., 1984). E. J. de Waal (2002) proposed an ADI of 0.1 μg/kg body weight, based on the NOEL of 0.1 mg/kg bw/day derived from the 90-day study in dogs by Nuehring et al. (1985) and an uncertainty factor of 1000. For a 70-kg adult, the ADI based on de Waal's argument would reached by consuming 50 g of corn per day containing CPA at a concentration of 0.14 μg/g.

## Discussion

As growers begin to use “atoxigenic” strains of *Aspergillus flavus* to reduce the accumulation of aflatoxin in maize, it is important for them to understand that certain “atoxigenic” strains may help to reduce aflatoxins but may not do anything to reduce CPA accumulation in grain or, in a worst case, may actually lead to higher levels of CPA in treated grain. The formulations and application timing for commercial “atoxigenic” strains are designed to allow the introduced strain to displace naturally occurring strains, and studies have shown that this happens with a high degree of success, with the introduced strains representing up to 96% of strains isolated from treated fields at the end of the crop season in which the “atoxigenic” strain was introduced and persisting at significant levels in the population over several years, even without retreatment (Cotty, 2006). With single or repeated use of AF36 or any similar CPA-producing strain, the overall population in a given field could include fewer aflatoxin producers but more CPA producers than the native population.

Georgianna et al. (2010) studied aflatoxin and CPA production by strains of *A. flavus* under 28 diverse conditions of nutrition and temperature and concluded that conditions favoring aflatoxin production, in general, also favor CPA production. In the past, it was perhaps reasonable to assume that steps aimed at keeping aflatoxin levels within acceptable limits would also keep the levels of CPA under control. The use of nonaflatoxigenic but CPA-producing strains like AF36 as crop protection products will decouple this passive control system for the first time and would potentially substitute one controlled risk for a different, but uncontrolled, risk. This is particularly concerning when the crop in question is maize as no other commodity plays such a major role in food and feed worldwide. Adding to this concern is the increasing use of maize for ethanol production, with the resulting dried distillers' grain and solubles (DDGS) being used for animal feed. Although specific data for CPA are not available, studies show that most mycotoxins are concentrated approximately threefold in the DDGS vs. the levels in raw maize (Bothast et al., 1992; Bennett and Richard, 1996; Murthy et al., 2005), and there is no reason to expect that this would differ in the case of CPA.

Sampling to determine CPA concentration in maize is subject to the same difficulties inherent in sampling for the presence of aflatoxin or any other mycotoxin, and this increases the uncertainty associated with analytical results and points to the need for robust sampling plans (Whitaker, 2006). The relative difficulty of the analytical methods for CPA also raises concerns. The relative weakness of the toxicological database and the more recent studies indicating potential for immunosuppression and organ damage at lower levels of exposure to CPA add uncertainty. Although there is now a small database for AF36 on maize, it is surely insufficient to provide any real assurance that elevated levels of CPA will not be observed in AF36-treated crops under certain conditions. As there is no routine monitoring for CPA levels, such elevated levels might only be discovered when animals or humans are harmed, as happened when undetected melamine contamination occurred in pet food in 2007, sickening and killing many dogs and cats before the cause could be identified and corrected (FDA, 2010). Unfortunately, the food/feed industry and public reaction to such an occurrence might not differentiate between AF36 and strains that do not produce CPA, and this could discredit the entire approach, robbing growers of one of the few tools they have to reduce mycotoxins in maize.

At a minimum, it is important that growers and other stakeholders are fully informed, in advance, of the potential risks so they can make informed choices. It would be far better for researchers, the crop protection industry, growers, aggregators, and processors to adopt clear minimum standards for any strain to be deployed in this manner, and these should include nonproduction of both aflatoxins and CPA and a robust genetic basis for nonproduction of these toxins. It is not difficult to identify strains that meet these criteria, and the agricultural community, including growers, aggregators, livestock and poultry producers, dairy farmers, and food and feed processors, should insist that all commercialized strains do so.
